# The geometry of evolutionary conflict

**DOI:** 10.1098/rspb.2022.2423

**Published:** 2023-02-08

**Authors:** Petri Rautiala, Andy Gardner

**Affiliations:** School of Biology, University of St Andrews, Greenside Place, St Andrews KY16 9TH, UK

**Keywords:** conflict, cost of complexity, Fisher's geometric model, major transitions, maladaptation, modularity

## Abstract

Conflicts of interest abound not only in human affairs but also in the biological realm. Evolutionary conflict occurs over multiple scales of biological organization, from genetic outlawry within genomes, to sibling rivalry within nuclear families, to collective-action disputes within societies. However, achieving a general understanding of the dynamics and consequences of evolutionary conflict remains an outstanding challenge. Here, we show that a development of R. A. Fisher's classic ‘geometric model’ of adaptation yields novel and surprising insights into the dynamics of evolutionary conflict and resulting maladaptation, including the discoveries that: (i) conflict can drive evolving traits arbitrarily far away from all parties' optima and, indeed, if all mutations are equally likely then contested traits are more often than not driven outwith the zone of actual conflict (hyper-maladaptation); (ii) evolutionary conflicts drive persistent maladaptation of orthogonal, non-contested traits (para-maladaptation); and (iii) modular design greatly ameliorates conflict-driven maladaptation, thereby facilitating major transitions in individuality.

## 1. Background

**Figure 1.**
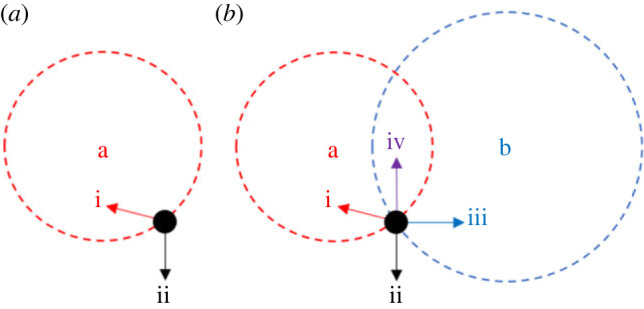
Fisher's geometric model of adaptation, extended to encompass conflicts of interest. (*a*) In the original formulation of Fisher's model [4], a mutation is beneficial (i) if it takes the phenotype (disc) closer to the optimum (**a**), and is deleterious (ii) if it takes the phenotype further from the optimum. (*b*) In the present formulation, there are two parties with two distinct optima (**a** and **b**), and so a mutation may be beneficial for one party but deleterious for the other (i and iii), beneficial for both parties (iv) or deleterious for both parties (ii).

Organisms are classically viewed as striving to maximize their fitness, with natural selection adaptively optimizing all their traits according to this purpose [1,2]. Here, a trait is any aspect of the world that is under the individual's control, whether it be part of the individual's body or behaviour, or an artefact like a spider's web, with the sum of all traits defining the individual's phenotype [3]. A simple, yet powerful, approach to investigating the dynamics of evolutionary adaptation was introduced by Fisher [1] in 1930. His geometric model of adaptation conceptualizes the phenotype at any moment in time as a point in multi-dimensional trait space, identifies the optimal phenotype as a different point in this multi-dimensional space and interprets mutation as a random leap in phenotype space, with the mutant phenotype being beneficial—and therefore supplanting the resident phenotype—if it lies closer to the optimum (figure 1*a*). Consideration of the resulting evolutionary dynamics has yielded detailed predictions concerning many aspects of the adaptive process, which often enjoy a strong quantitative fit with empirical data [4–14].

However, in many situations the evolutionary interests of the individual may come into conflict with those of other individuals and, indeed, with the interests of other biological entities. Individuals may be embroiled in evolutionary conflicts with their mates [15,16], offspring [17], parents [17], siblings [18] and other social partners [19], including those belonging to other species [20,21]. In addition, the interests of a whole social group may be in conflict with those of its constituent members, as in the case of public-goods dilemmas and the tragedy of the commons [22]. And, within an individual's genome, different genes may come into conflict with the individual and with each other, for example owing to differences in mode of transmission [23–25]. These evolutionary conflicts clearly pose a barrier to adaptation, and conflict-driven maladaptation has been implicated in a wide range of human pathologies, including growth and fertility disorders and childhood cancers [26–28].

## 2. Results

To investigate the evolutionary dynamics of adaptation and maladaptation in the context of conflicts of interest, we alter a key assumption of Fisher's geometric model to consider that the phenotype is controlled by more than one biological agent and that these agents have different agendas (figure 1*b*). Specifically, we focus on scenarios in which there are two different optima, **a** and **b**, in the multi-dimensional trait space, each corresponding to a different agent (or bloc of agents) that exerts some control over the phenotype. We assume that every point in space is mutationally connected to every other, and that with an independent probability *p* for each mutation, this mutation is considered to be governed by optimum **a**, such that it is beneficial if and only if it brings the phenotype closer to optimum **a**, and with probability 1 − *p* the mutation is instead governed by optimum **b**, such that it is beneficial if and only if it brings the phenotype closer to optimum **b**. We assume that the phenotype space is finite, but allow this to be of arbitrarily large size (see electronic supplementary material, §S1 for full details).

**Figure 2.**
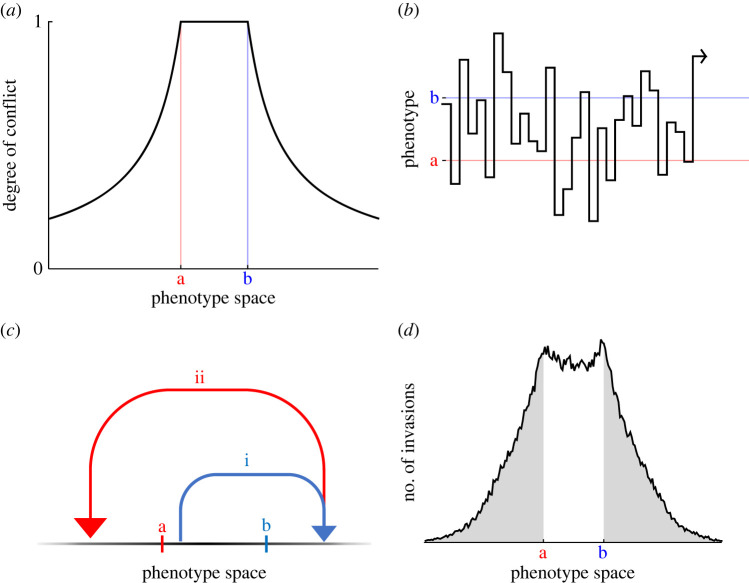
Evolutionary dynamics in one dimension and conflict-driven maladaptation. (*a*) The degree of conflict is maximal between the two optima and declines monotonically outwith this interval. (*b*) Conflict drives never-ending fluctuation in the phenotype both within and outwith the interval between the two optima (shown here is a single numerical simulation run for 30 invasion events). (*c*) The phenotype can escape the interval between the optima by leap-frogging between the far sides of each optimum, to become arbitrarily distant from either. (*d*) At equilibrium, a probability density emerges in which the phenotype is at least as likely to be found outwith the interval between the two optima as within it (shown here is a single numerical simulation run for 1.5 × 10^5^ invasion events). For the purpose of illustration, (*a*,*b*,*d*) assume that all mutations are equally likely and (*b,d*) make use of Fisher's assumption that beneficial alleles are certain to proceed to fixation (see electronic supplementary material, §§S1.3 and S2 for full details).

This geometric approach allows us to explicitly quantify the intensity of evolutionary conflict at any point in the multi-dimensional trait space, which we define as the probability that the next beneficial mutation—whether it be governed by optimum **a** or optimum **b**—is beneficial only for the governing agent and is deleterious for the other agent. We find that the intensity of conflict is maximal and equal to 1 when the phenotype lies on the line segment between the two optima, because here all mutations that take the phenotype closer to optimum **a** necessarily move it further away from optimum **b**, and *vice versa*, and the intensity of conflict is lower for all points in trait space outwith this line segment, as here it is possible for a mutation that takes the phenotype closer to optimum **a** to also take the phenotype closer to optimum **b**, and *vice versa* (figure 2*a*; see electronic supplementary material, §S2).

In addition to quantifying conflict, our model enables us to explicitly track the evolutionary dynamics of the conflicted phenotype. In contrast with Fisher's classic single-optimum formulation of the geometric model, in which the phenotype moves progressively closer to the optimum with each mutation-invasion event, the conflict of interests in our two-optima version of the geometric model leads to persistent maladaptation and sustained phenotypic fluctuation (figure 2*b*; see electronic supplementary material, §S3). Specifically, as the phenotype moves closer to one fitness optimum, a subsequent mutation governed by the other optimum is liable to move the phenotype away in the other direction, such that the phenotype never settles upon a fixed evolutionary endpoint.

Moreover, within the context of a one-dimensional phenotype space, while it might be supposed that the phenotype would oscillate within the ‘battleground’ [29] interval bounded by the two optima, we find instead that the conflict of interests frequently leads to the phenotype being driven outwith this interval, representing ‘hyper-maladaptation’ that is in neither party's evolutionary interest. The phenotype can escape from the battleground interval because this mutational step may bring it closer to one of the optima, and the distance from either optimum may be increased to an arbitrarily large extent by a succession of mutations that ‘leap-frog’ from the far side of one optimum to the far side of the other, and so on (figure 2*c*; see electronic supplementary material, §S3). Indeed, if all mutations are equally likely, then irrespective of the location of the current phenotype or the balance of power between the conflicting agents, the next beneficial mutation is at least as likely to yield a phenotype that lies outwith the zone of conflict as it is to yield a phenotype that lies between the two optima (figure 2*d*; see electronic supplementary material, §S3).

**Figure 3.**
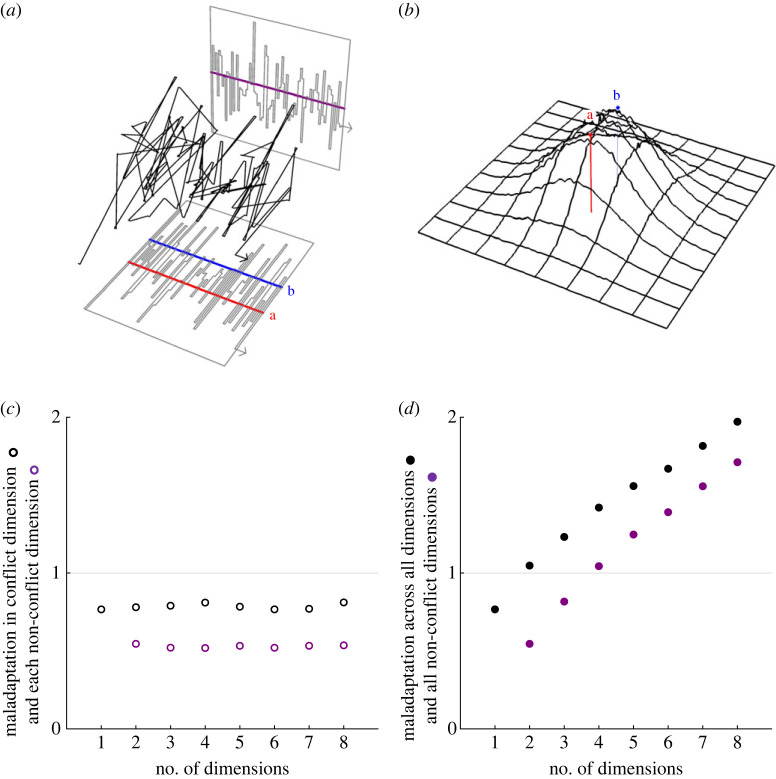
Evolutionary dynamics in multiple dimensions and the conflict-driven cost of complexity. (*a*) Conflict drives never-ending fluctuation in the phenotype in both conflicted and non-conflicted traits, resulting in substantial sub-optimality, including in traits for which there is no actual conflict (shown here is a single numerical simulation run in two dimensions for 40 invasion events). (*b*) At equilibrium, there is substantial maladaptation even in trait dimensions in which conflict does not exist (shown here is the probability density of invasion events corresponding to a single numerical simulation run in two dimensions for 3 million invasion events). (*c*) The extent of maladaptation in each dimension is approximately constant with respect to the dimensionality of the phenotype space (each dot corresponds to the endpoint after 100 invasion events, averaged over 500 numerical simulation replicates; measurements are in units of the distance between optima). (*d*) Accordingly, the overall maladaptation in all dimensions scales approximately with the square root of the dimensionality of the phenotype space, such that conflict induces a cost of complexity that inhibits precision fine-tuning of complex adaptation (each dot corresponds to the endpoint after 100 invasion events, averaged over 500 numerical simulation replicates; measurements are in units of the distance between optima). For the purpose of illustration, (*a*)–(*d*) assume that all mutations are equally likely and also make use of Fisher's assumption that beneficial alleles are certain to proceed to fixation (see electronic supplementary material, §§S1.3 and S5 for full details).

Furthermore, when there is more than one trait dimension, we find that the conflict of interests spills over from the conflicted trait to drive persistent maladaptation in orthogonal traits with respect to which no conflict actually exists (figure 3*a*,*b*; see electronic supplementary material, §S3). If all mutations are equally likely, then in the long run the extent of such ‘para-maladaptation’ in each orthogonal dimension is directly proportional to the distance between the two optima within the conflict dimension, and we find that the degree of maladaptation—i.e. the average Euclidean distance of the phenotype from the governing optimum—within each dimension is approximately constant with respect to the dimensionality of the phenotype space (figure 3*c*). As a consequence of this, the overall degree of maladaptation—i.e. the average Euclidean distance of the phenotype from the governing optimum across all dimensions simultaneously—increases with the dimensionality of the phenotype space (in particular, it scales approximately with the square root of the number of phenotypic dimensions; figure 3*d*). This constitutes a conflict-driven cost of complexity that is absent in Fisher's original model. Even though the conflict arises in relation to a single phenotypic dimension, its maladaptive consequences are magnified as the phenotype becomes more complex, such that conflicts pose a strong barrier to precision fine-tuning of complex adaptations.

**Figure 4.**
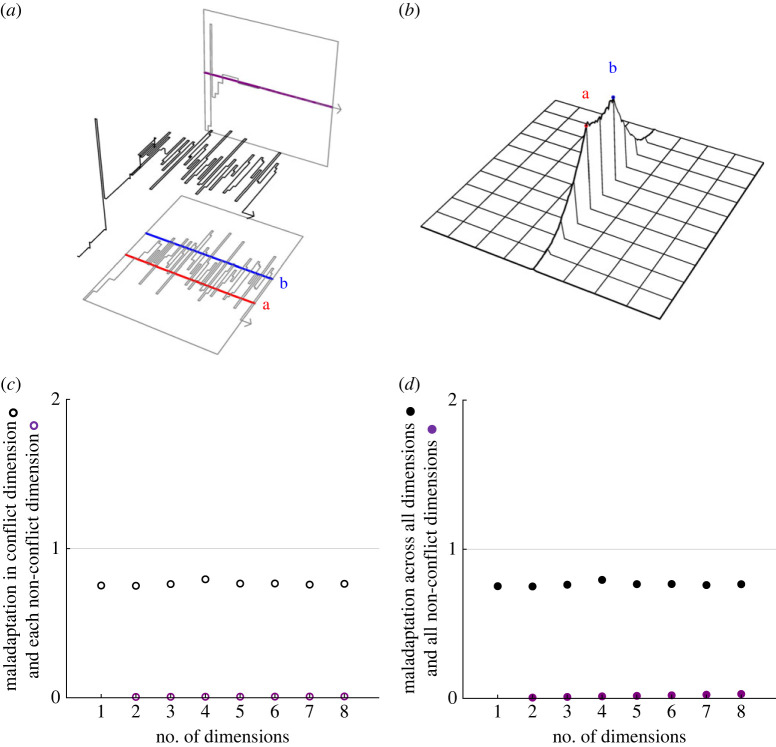
Modularity abolishes the conflict-driven cost of complexity. (*a*) Although conflict drives persistent maladaptation of conflicted traits, modularity ensures precision fine-tuning of non-conflicted traits that are underpinned by different mutations (shown here is a single numerical simulation run in two dimensions for 40 invasion events). (*b*) This results in a vanishingly low probability of the phenotype occurring outwith the conflict dimension at equilibrium (shown here is a single numerical simulation run in two dimensions for 1.5 × 10^5^ invasion events). (*c*,*d*) Accordingly, the extent of maladaptation tends to zero in the long run within all trait dimensions that are free of conflict, resulting in an overall degree of maladaptation across all dimensions that is approximately constant with respect to the dimensionality of the phenotype space, such that evolutionary conflicts cease to induce a cost of complexity in the presence of modularity (each dot corresponds to the endpoint after 100 invasion events, averaged over 500 numerical simulation replicates; measurements are in units of the distance between optima). For the purpose of illustration, (*a*)–(*d*) assume that all mutations are equally likely and also make use of Fisher's assumption that beneficial alleles are certain to proceed to fixation (see electronic supplementary material, §§S1.3 and S6 for full details).

This conflict-driven para-maladaptation results from the assumption that single-mutational events may simultaneously affect multiple traits. Such pleiotropy is prevalent in Fisher's classic formulation of the geometric model. The opposite of pleiotropy is modularity whereby, in its extreme form, each mutation represents a phenotypic transformation along a single-trait axis without perturbing the location of the phenotype along any other axis [14]. Incorporating mutational modularity into our model, we find that the phenotype moves monotonically closer to the optimum in every unconflicted trait dimension, such that in the long run these dimensions achieve optimality and can be discarded from the analysis of evolutionary dynamics (figure 4*a*; see electronic supplementary material, §S6). This means that the equilibrium state exactly recovers that of a lower-dimensionality model that neglects the unconflicted trait dimensions (figure 4*b*). Consequently, we find that modularity abolishes the conflict-driven cost of complexity and thereby facilitates complex adaptation (figure 4*c*,*d*).

The extensive maladaptation associated with evolutionary conflict may mean that both parties are worse off on average than they would have been had they compromised and agreed to settle on the same mutually sub-optimal phenotype, in a way that is reminiscent of mutually deleterious outcomes in the theory of games [30,31]. In the pleiotropy scenario considered above, assuming all mutations are equally likely and that the power of the two agents is equally balanced, we find that the expected distance of the phenotype from each party's optimum is always greater than half the distance between the two optima (figure 3*d*), meaning that both parties would be better off splitting the difference and agreeing to aim for the midpoint between their two optima rather than persist in engaging in mutually deleterious conflict. And, for all scenarios with dimensionality 2 or higher, the expected distance of the phenotype from each party's optimum is actually greater than the distance between the two optima (figure 3*d*), such that each party would actually be better off allowing the phenotype to go to their adversary's optimum than to engage in the conflict. However, these results concern the expected degree of maladaptation that emerges in the long run, and in the short term neither party is favoured to relinquish control of the phenotype as this would always tend to make them worse off with respect to the very next evolutionary step (see electronic supplementary material, §S9).

So far, we have assumed that an agent considers a mutation to be beneficial if it brings the phenotype closer to their optimum. However, our framework readily extends to scenarios in which the agent considers a mutation to be beneficial if it takes the phenotype further away from their pessimum—this being the point in trait space at which the agent's agenda is most poorly realized [1]. In the non-conflict scenario with only one agent and one corresponding pessimum, which was briefly considered by Fisher [1], the probability of the phenotype remaining close to the pessimum trivially tends to zero as time tends to infinity. By contrast, in the conflict scenario with two agents having different pessima, there remains a substantial probability of the phenotype remaining close to each pessimum in the long run, provided that the two agents also disagree as to which is the least-worst phenotype (see electronic supplementary material, §§S7 and 8).

## 3. Discussion

We have shown that evolutionary conflicts of interest pose a major barrier to the process of adaptation. Conflicts of interest not only destroy the asymptotic approach towards adaptive perfection described by Fisher's original formulation of the geometric model of adaptation, but may also drive the phenotype outwith the battleground into realms that are mutually agreed to be deleterious to fitness, including into orthogonal phenotypic dimensions within which no conflict actually exists. These results contrast with those of a recent simulation analysis [32] of a similar model, inspired by host–pathogen coevolution, in which the phenotype space was limited to the one-dimensional interval between the two optima, which understated the scope for hyper-maladaptation and para-maladaptation. Our finding regarding the propensity for conflict to drive maladaptation in relation to non-conflicted traits has been previously emphasized by Wilkins [33], but for a different reason: his model assumes particular pleiotropic relationships between traits such that optimization in one trait dimension necessarily drives maladaptation in others, whereas our model requires no such assumption and has para-maladaptation arising for purely statistical reasons. We have described a new conflict-driven cost of complexity and have shown that this increases with the dimensionality of the phenotype space, analogous to how higher dimensionality is expected to impede adaptation in non-conflict scenarios, including in terms of slowness of approach to a single static optimum in Fisher's original formulation [1,7] and also in terms of persistent maladaptation as a result of lineages experiencing a perpetually shifting optimum owing to either environmental change [34] or migration through a spatially heterogeneous environment [35].

Our analysis pertains to true conflicts, whereby one optimum governs the evolutionary trajectories of some mutations and another optimum governs those of others—as for example when maternally expressed genes at some imprinted loci have different fitness interests from paternally expressed genes at other imprinted loci [24]. Scenarios in which the same mutation moves between different selective environments, and hence has an optimum that represents an averaging of the selection pressures experienced across these different environments—as for example when a sexually antagonistic allele [36] finds itself carried sometimes by females and sometimes by males—are occasionally referred to in terms of ‘conflict’ but may be better conceptualized as simply involving trade-offs [25]. For the purpose of describing equilibrium states, we have considered snap-shots of the population at the moment at which new beneficial mutations invade, which is appropriate if making predictions about the archaeological record of evolutionary history laid down in the genome, rather than during the intervals between these invasion events, which would be appropriate if making predictions as to current phenotypes in contemporary populations. However, this distinction does not qualitatively affect any of our results (see electronic supplementary material). For simplicity, we have focused on the extremes of full pleiotropy (each mutation has random effects in all dimensions) versus full modularity (each mutation has a random effect in only one dimension), though intermediate scenarios (correlations in mutational effects across dimensions) [37,38] might also be of interest. Finally, a key assumption of our analysis has been that there are only two conflicting agents, and extension to three or more adaptive agents—including with the potential for coalition formation—represents an important avenue for future investigation.

Our model allows for asymmetries in agent control over the phenotype, by specifying that each mutation that arises has an independent probability *p* of being governed by one agent's agenda and probability 1 − *p* of being governed by the other agent's agenda. Numerical investigation suggests that the average degree of maladaptation is maximal when both agents exert equal control over the phenotype (i.e. *p* = ½) and declines monotonically as one agent increasingly controls the phenotype (i.e. *p* declines towards 0 or increases towards 1; see electronic supplementary material, §S10). Accordingly, we might expect conflicts to be particularly damaging—and hence of biological and clinical importance—when they occur between roughly evenly matched opponents, such as maternal-origin genes and paternal-origin genes at imprinted loci, which will in many cases be equally numerous within the genome, and this might go some way to explaining the severity of many imprinting-related disorders [26–28].

Our analysis prompts a rethink of current understanding on the link between conflicts of interest and major transitions in evolution. It has long been recognized that a mismatch of optima limits the ability for social groups to emerge as higher levels of individuality in their own right, as for example in the evolution of social-insect colony ‘superorganisms’ [39–41]. This has led to the suggestion that, where such transitions have occurred, the superorganism represents only that portion of the social group's phenotype that is free from conflict, with the remainder of the phenotype being of a non-superorganismal nature [42,43]. For example, a social-insect superorganism is understood to include the aggregate of the colony's ‘resource acquisition’ traits, such as cooperative foraging, over which there is expected to be little or no conflict, and to exclude the colony's ‘resource allocation’ traits, such as differential feeding of male versus female brood, over which strong intra-colony conflict may occur [42,43]. However, we have shown that conflict may drive maladaptation even within conflict-free trait dimensions, reducing the scope for superorganismality to emerge. We have also shown that mutational modularity may provide a solution to this problem, enabling optimization of conflict-free traits independently of those for which there is a mismatch between optima. Accordingly, we suggest that modularity is a crucial—and previously overlooked—enabler of major transitions in evolution.

Finally, our analysis provides new conceptual support for the principle of the ‘veil of ignorance’ as a promoter of harmony within genomes and within insect and human societies [44–48]. In particular, the suppression of information concerning kin relationships between social partners has been argued to boost overall levels of cooperation, but a recent analysis [49] has shown that—all else being equal—this veil of ignorance is just as capable of inhibiting cooperation or having no impact at all. To the extent that such kinship information modulates an agent's agenda, as for example when information about a gene's parent-of-origin brings it into conflict with its homologue in a way that would not occur if both genes were ignorant of their parent-of-origin, then suppression of this kinship information would tend to cause the different optima to converge upon the same intermediate trade-off. Our analysis reveals that, even if granting such information to each agent and allowing them the opportunity to move the phenotype towards their own divergent optimum could potentially result in a higher fitness payoff, the maladaptation arising from the ensuing conflict dynamics may be so damaging as to mean that all agents would be better off under the veil of ignorance.

## Data Availability

All code is available from Dryad (https://doi.org/10.5061/dryad.4f4qrfjgn) [50]. The data are provided in the electronic supplementary material [51].
